# Turmeric supplementation improves markers of recovery in elite male footballers: a pilot study

**DOI:** 10.3389/fnut.2023.1175622

**Published:** 2023-05-24

**Authors:** David J. Clayton, Ross Burbeary, Philip J. Hennis, Ruth M. James, Christopher Saward, Amy Colledge, Reece Scott, Steve Gilpin, Ryan McMahon, Ian Varley

**Affiliations:** ^1^Musculoskeletal Research Group, School of Science and Technology, Nottingham Trent University, Nottingham, United Kingdom; ^2^Derby County Football Club, Pride Park Stadium, Derby, United Kingdom; ^3^Rotherham United Football Club, AESSEAL New York Stadium, Rotherham, United Kingdom

**Keywords:** curcumin, polyphenols, elite athlete, recovery, football (soccer), inflamation

## Abstract

Football match-play causes muscle damage and provokes an inflammatory response. Rapid recovery is paramount to optimising subsequent performance and reducing injury risk. Turmeric contains high concentrations of curcumin, a polyphenol that has been shown to reduce muscle damage and soreness post-exercise in recreational exercisers. However, it is unknown whether a curcumin-containing supplement can support elite footballers recovery between matches. This applied study explored whether a turmeric supplement could improve performance, subjective and physiological markers of recovery, in elite male footballers. Twenty-four elite male footballers divided into a turmeric group, who consumed 60 mL of a turmeric drink twice per day, or a control group who did not. After 96 h of rest, baseline measurements of subjective soreness (leg and whole-body), plasma creatine kinase ([CK]), plasma C-reactive protein ([CRP]), isometric mid-thigh pull (IMTP) and counter movement jump (CMJ), were collected. Following eight competitive matches, subjective leg and whole-body soreness and plasma concentrations of inflammation markers ([CK] and [CRP]) were assessed immediately (0 h), 40 and 64 h post-match. Performance markers (IMTP and CMJ) were also assessed at 40 and 64 h post-match. Percentage change from baseline showed a main effect of group (*p* = 0.035, *p* = 0.005) and time (*p* = 0.002, p = 0.002) for both leg and whole-body soreness, respectively. There was a group by time interaction effect (*p* = 0.049) for [CRP]. There were no effects of turmeric on [CK], CMJ or IMTP. This applied study is the first in elite footballers to show that a curcumin-containing supplementation may attenuate a biomarker of inflammation [CRP] and soreness post-match play.

## Introduction

Prolonged, high-intensity exercise causes muscle damage and produces reactive oxygen species, provoking an inflammatory response and altering cell function (1). Whilst inflammation is a necessary part of the tissue repair process (1), this often leads to muscular discomfort and a subsequent reduction in the ability to generate force (2). Following muscle damaging exercise, limitations of the endogenous antioxidant system to remove free-radicals delays recovery and reduces subsequent exercise performance in a time-course manner often referred to as delayed onset of muscle soreness (DOMS) (2).

Professional footballers in the UK are subject to a high number of competitive matches (>50 per season) and often short intervals between matches (48–72 h), which curtails recovery time and increases the risk of injury or sub-optimal performance (3). Football match-play is known to cause a significant increase in muscle damage and soreness up to 72 h post-match (4, 5), likely due to the high magnitude eccentric muscle loading (6). Professional footballers often use non-steroidal anti-inflammatory drugs (NSAIDs) to alleviate DOMS and restore muscle function (7). However, concerns about the long-term use of NSAIDs and reported side effects, such as reduced muscle regeneration (7), and gastrointestinal problems (8) necessitates the need for alternative solutions (7).

Supplementation of certain dietary compounds may aid recovery by reducing inflammation, alleviating DOMS and restoring muscle function (9). Curcumin, a natural polyphenol found in high concentrations in turmeric, has anti-inflammatory, antioxidant, and analgesic properties, making it a candidate to accelerate post-exercise recovery. Laboratory-based studies of cycling, running and eccentric loading protocols (e.g., downhill running, eccentric resistance exercises), have shown that curcumin supplementation before and after an exercise period can reduce subjective soreness (10, 11), attenuate haematological inflammatory markers (11), and improve subsequent exercise performance (12, 13). However, there is an absence of applied studies in elite cohorts, likely due, in part, to challenges associated with conducting research in elite cohorts. One study, in elite rugby players, found that curcumin attenuated muscle damage and limited loss of muscle function after a muscle damaging protocol (12), while one other study in youth team male footballers found that curcumin attenuated DOMS and loss of muscle function after match-play (14). However, no study has explored whether a curcumin supplement can accelerate recovery from match-play in elite-level footballers. Elite sport is unique in terms of the playing intensity and specific contextual factors such as athlete skill level, psychological pressure and playing environment (crowd, score line, etc.), which make it hard to replicate in laboratory-based studies and/or with non-elite participants (15).

Whilst early findings are encouraging (15), whether curcumin can accelerate recovery and more rapidly restore muscle function in elite athletes is not well known. The aim of this study was to assesses if regular turmeric supplementation could accelerate markers of recovery, including, haematological inflammatory markers, subjective soreness, and performance, in elite male footballers following match-play. Participants consumed a commercially available turmeric supplement, formulated to contain 35 g of raw turmeric root with an estimated curcumin content of 1,400 mg (16), twice a day throughout the course of the study.

## Methods

### Participants

Twenty-four elite [Tier 4; (17)] male professional footballers (with 7.5 ± 3.7y experience as a professional footballer and undertaking 3–4 training sessions per week), competing in the English third tier during the 2020/21 season, volunteered to participate in the study. To qualify for enrolment, participants were required to be outfield players with no history of cardiovascular or gastrointestinal complaint and were not regularly consuming any supplements containing turmeric or curcumin. All participants provided written consent after they were informed verbally and in writing of the nature and requirements of the study. The study was approved by the Nottingham Trent University Human Invasive Ethics Committee (REF: 716).

### Study design and measures

The study adopted a between-groups design. Baseline measures of subjective soreness (whole-body and leg-specific), performance [counter movement jump (CMJ) and isometric mid-thigh pull (IMTP)] and concentration of haematological markers of inflammation [C-reactive protein ([CRP]) and creatine kinase ([CK]) were collected after 96 h of rest. Following this, participants self-selected to a supplementation or control group. The supplementation group (n = 16; Age = 26 ± 3 y; Height = 1.83 ± 0.06 m; Mass = 80.2 ± 5.0 kg) consumed two 60 mL drinks per day (Raw Turmeric Original Shot, The Turmeric Co., UK)], each containing 35 g of raw turmeric root (estimated to contain 1,400 mg curcumin) and 200 mg of black pepper (estimated to contain 10 mg of piperine), throughout the course of the study. Compliance with the supplementation was verbally confirmed by participants prior to each match. The control group (n = 8, Age = 25 ± 4 y; Height = 1.82 ± 0.07 m; Mass = 79.5 ± 6.3 kg) were not provided with the supplement and were asked to avoid turmeric-containing supplements during the study. Participants were monitored during eight competitive matches taking place between October 2021 and April 2022 using Global Positing System (Vector, Catapult, Australia) for commonly used physical performance measures (total distance, high speed distance, accelerations, deceleration). There were no differences in GPS derived physical performance measures between the turmeric group and the control group (all *p* values <0.29; for data see Supplementary File 1). Participants were assessed immediately after each match for measures of subjective soreness and inflammation, and these were assessed again, along with performance measures, 40 h and 64 h following each match.

### Procedures

Capillary blood samples (300 μL) were collected into EDTA collection tubes (Microvette, Sarstedt, Germany). Post-match samples were collected where the match took place, with follow-up samples collected on arrival at the training facility. Samples were stored on ice after collection (maximum of 30 min) before being centrifuged (13,000 *g*, 10 min, 4°C), and the resulting plasma being stored at −80°C. Plasma samples were analysed for [CK] and [CRP] using an ABX Pentra 400 (Horiba Medical, Kyoto, Japan; CV: 3.2%).

Subjective whole-body and leg-specific soreness were assessed on 100 mm visual analogue scales, anchored at 0 mm with ‘no pain’ and 100 mm with ‘as much pain as it could be’ (4, 18). Participants were asked to apply a mark on the line to indicate their level of soreness, which was then quantified with a ruler.

CMJ was assessed by participants standing in an upright position with their hands on their hips, squatting to their preferred depth, before jumping as high as possible whilst standing on a force plate (Hawkin Dynamics, ME, United States). Jump height was calculated using the following equation: Jump Height = Take Off Velocity^2^/2 x Gravity (Moir 2008). CMJs have been shown to be reliable [ICCs 0.96–0.97; (19)]. Participants prepared with an individualised warm-up followed by three jumps, with the highest jump recorded and used in subsequent analysis. Verbal encouragement was provided by the investigators for each jump.

The IMTP test was performed on two force platforms sampling at 1000 HZ (Hawkin Dynamics, ME, United States) and a test rack. The IMTP has been shown to be a reliable test [ICCs 0.89–0.98 (20)]. Participants used a standardised overhand grip while secured to the barbell with lifting straps to eliminate the influence of grip strength. Warm-up trials were performed prior to completing three maximal lifts of 3 s per lift. Verbal encouragement was provided by the investigators for each lift. Peak force and percentage change in peak force were used for the statistical analysis.

### GPS

The physical demands for match-play were monitored using a 10 Hz GPS (Vector, Catapult, Australia). This system has been valid and reliable to assess athlete movements (21). Each player wore a bespoke garment containing a GPS unit positioned between the shoulder blades. Post-session, each GPS unit was downloaded and analysed using commercially available software (Viper, STATSports, Ireland). The physical performance variables assessed included: total distance covered (m), high speed (>5.5 m/s) distance covered (m), very high speed (>7.0 m/s) distance covered (m), number of accelerations above 0.5 m/s^2^ for >0.5 s, and number of decelerations below −0.5 m/s^2^ for >0.5 s (Supplementary Table 1).

### Statistical analysis

Two-way repeated measures analysis of variance (ANOVA) were conducted on each recovery marker with percentage change from baseline serving as the dependent variable and time and supplementation group as the independent variables. Where appropriate, significant main effects were followed-up with Bonferroni-corrected post-hoc tests. Statistical significance was accepted at the 95% confidence level (*p* < 0.05). Mean and standard error were used to describe the average and variability of data, unless stated otherwise. Partial eta squared statistics were used to indicate effect sizes and were interpreted as small (0.01–0.05), medium (0.06–0.13), and large (≥0.14) (22). Analyses were conducted using IBM SPSS Statistics (v.28).

## Results

### Markers of inflammation

**Figure 1:**
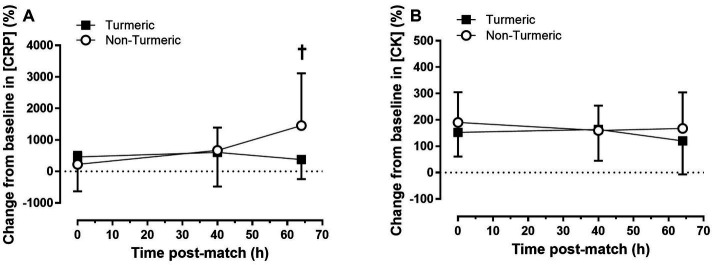
Plasma concentrations of c-reactive protein **(A)** and creatine kinase **(B)** immediately (0 h), 40 h and 64 h post-match, in the turmeric (solid square) and non-turmeric (open circle) groups. † indicates a difference between groups at this time-point (*p* < 0.05); Data are presented as percent change from baseline. Values are means with error bars representing standard error.

There was no main effect of time (*p =* 0.08, η^2^_p_ = 0.034) or group (*p* = 0.13, η^2^_p_ = 0.015) for percentage change in plasma [CRP] but there was a significant group by time interaction (*p* = 0.049, η^2^_p_ = 0.040; see Figure 1A). At 64 h post-match, [CRP] was 1,082 ± 447% lower in the turmeric group compared to the control group (*p* = 0.017). There was no main effect of time (*p* = 0.64, η^2^_p_ = 0.005) or group (*p* = 0.23, η^2^_p_ = 0.008), and no group by time interaction (*p* = 0.56, η^2^_p_ = 0.006) for percentage change in plasma [CK] (see Figure 1B).

### Subjective markers of soreness

**Figure 2:**
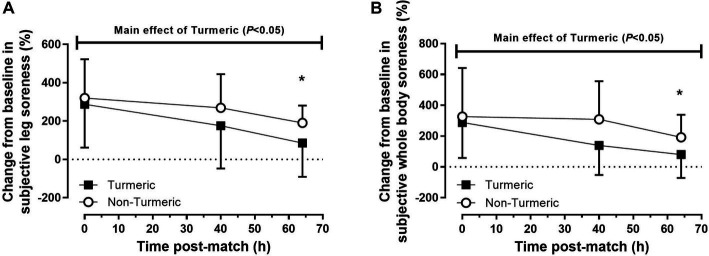
Subjective ratings of leg **(A)** and whole-body **(B)** soreness immediately (0 h), 40 h and 64 h post-match, in the turmeric (solid square) and non-turmeric (open circle) groups. * indicates a difference compared to 0 h in both groups (*p* < 0.05). Data are presented as percent change from baseline. Values are means with error bars representing standard error.

The percentage change in leg soreness was 77 ± 36% lower overall in the turmeric group compared to the control group (*p* = 0.035, η^2^_p_ = 0.022). There was also a main effect of time (*p* = 0.002, η^2^_p_ = 0.06), for percentage change in leg soreness where post-hoc analysis revealed that leg soreness was 165 ± 46% lower in both groups at 64 h compared to 0 h post-match (*p* < 0.001), but there was no difference between 0 and 40 h (*p* = 0.15) or 40 and 64 h (*p* = 0.21) post-match. There was no group by time interaction effect for leg soreness (*p* = 0.66, η^2^_p_ = 0.004; see Figure 2A).

Whole-body soreness was 106 ± 37% lower overall in the turmeric group compared to the control group (*p* = 0.005, η^2^_p_ = 0.038). There was also a main effect of time for whole-body soreness (*p* = 0.002, η^2^_p_ = 0.061) where post-hoc analysis revealed that soreness was lower in both groups at 64 h compared to 0 h post-match (*p* = 0.001; Figure 2B), but there were no differences between 0 and 40 h (*p* = 0.14) or 40 and 64 h (*p* = 0.19) post-match. There was no group by time interaction effect for whole-body soreness (*p* = 0.29, η^2^_p_ = 0.012).

### Performance markers

**Figure 3:**
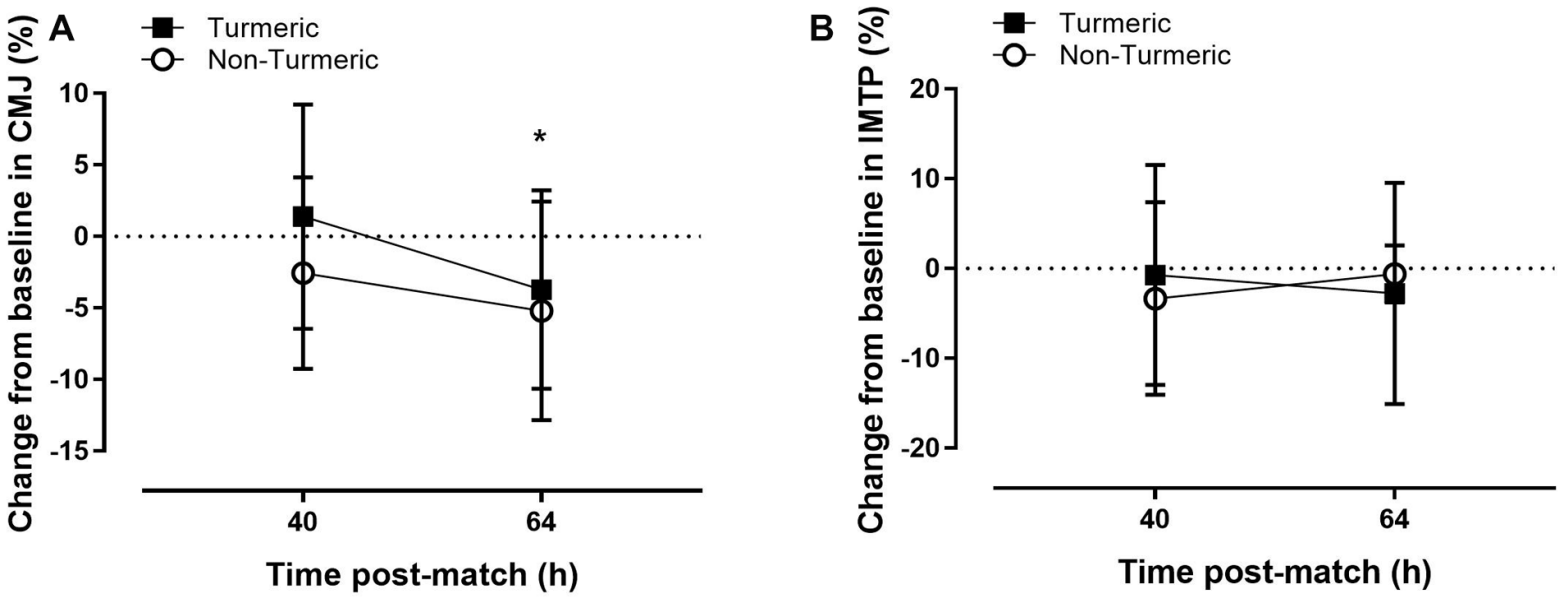
Countermovement jump height **(A)** and isometric mid-thigh pull **(B)** performance 40 h and 64 h post-match, in the turmeric (solid square) and non-turmeric (open circle) groups. * indicates a difference compared to 40 h in both groups (*p* < 0.05). Data are presented as percent change from baseline. Values are means with error bars representing standard error.

CMJ performance was 3.9 ± 1.9% lower at 64 h compared to 40 h post-match (*p* = 0.049, η^2^_p_ = 0.052). There was no main group (*p* = 0.16, η^2^_p_ = 0.026) or group by time interaction (*p* = 0.53, η^2^_p_ = 0.006) effects for percentage change in CMJ (see Figure 3A). There were no main effects of time (*p* = 0.93, η^2^_p_ < 0.001), group (*p* = 0.95, η ^2^_p_ < 0.001), nor group by time interaction (*p* = 0.54, η ^2^_p_ < 0.006) for percentage change in IMTP (see Figure 3B).

## Discussion

The aim of this study was to determine if twice daily consumption of a commercially available turmeric supplement, estimated to contain 1,400 mg of curcumin, could enhance recovery in a group of elite male footballers. We found that turmeric supplementation attenuated subjective markers of muscle soreness and reduced plasma [CRP] – a haematological marker of inflammation – at 64 h post-match, compared to a control group who did not consume the supplement. The results of this pilot study provide evidence that a commercially available turmeric supplement may be an effective and convenient method of accelerating recovery in elite male footballers.

Increased plasma [CRP] was observed in the control group 64 h post-match, but was attenuated in the turmeric supplementation group, indicating significant blunting of the systemic inflammatory response in participants consuming the turmeric supplement. CRP is a major acute phase protein synthesised by the liver in response to elevations in IL-6 and correlates with systemic inflammation (23). Turmeric contains high levels of the polyphenol curcumin, which has antioxidant and anti-inflammatory properties (24). Previous studies demonstrate that curcumin downregulates various inflammatory regulators, including nuclear factor kappa beta (NF-κB) activation and production of the enzyme cyclooxygenase-2 (COX-2), which are central components of the inflammatory cascade (11). Curcumin also increases blood antioxidant capacity, reducing the inflammation caused by free radicals (25). Therefore, the reduction in [CRP] at 64 h post-match likely reflects a reduction in systematic inflammation, indicating that turmeric supplementation has attenuated exercise-induced inflammation.

In contrast to [CRP], plasma [CK] did not differ between groups. CK is a protein found in the mitochondria of the muscle and can be used as a biomarker for muscle damage (26). The results of the present study are similar to another supplementation study, which found after an acute bout of eccentric bicep exercise, that [CRP] could be reduced by an antioxidant-docohexanoic supplement, but [CK] remained unchanged (27). Differences in [CK] and [CRP] response in the present study may be related to the training status of the population studied. Previous research has shown that plasma [CK] increases less in individuals accustomed to high volumes of exercise (28, 29) and can be influenced by muscle fibre type (30). Differences may also be due to the limited amount of control we were able to apply, due to the professional-status and elite nature of the population studied. For example, acute dietary intake of certain nutrients [such as vitamin C; (31)], which we were unable to measure or control in the present study, can influence plasma [CK]. Despite no club directed training taking place, it is also possible that the 5-days of rest prior to baseline measurements were insufficient for [CK] to reach nadir, which, coupled with these other factors, may explain why we did not observe a change in [CK] in response to turmeric supplementation.

Subjective muscle soreness was reduced with turmeric supplementation across all post-match time points. Previous studies have also similarly found that curcumin supplementation has reduced DOMS after exercise (10, 11), however, this finding is not consistent amongst all curcumin supplementation studies (12, 13, 32). Previous studies have ceased supplementation the day before (32) or 12 h after (13) exercise, which may explain the null findings. Tanabe et al. (33) found that curcumin supplementation for 4-days after exercise reduced muscle soreness, whereas supplementation for 7-days preceding exercise did not. These findings align with the current study and suggest that the analgesic effects of curcumin supplementation may be enhanced by continuing supplementation throughout the recovery period. This is likely explained by the pharmacokinetics of polyphenols, which typically peak a few hours after consumption before being metabolised and excreted (34).

Despite turmeric supplementation reducing CRP and subjective markers of soreness, this study found no effect of supplementation on CMJ and IMTP performance up to 48 h post-match. Previous studies have reported inconsistent findings regarding the effects of curcumin supplementation on performance, following a bout of exercise induced muscle damage. For example, curcumin supplementation can expedite recovery of some performance outcomes, such as MVC torque (13), range of motion (33) and 6 s sprint performance (12). However, other performance metrics, such at total work, mean peak torque (13) and MVC torque (33) were not different between when supplementing with curcumin. Abbott et al. (14) reported that curcumin supplementation offset the attenuation in CMJ performance observed after exercise-induced muscle damage from football match-play. These findings are directly comparable, in terms of exercise mode and performance metric, to the current study, yet present conflicting results. This may be due to the contextual differences in academy (14) compared to elite (current study) football match-play, and/or the degree of control that could be elicited around performance testing. For example, Abbott et al. (14) collected all measurements in the fasted state, whereas we were unable to control players dietary intake prior to measurements.

The supplement administered in this study was a 60 mL shot, which was well-tolerated and easy to incorporate into the nutrition plan of elite footballers. The supplement also contained piperine, an adjuvant that can greatly improve bioavailability of curcumin. High doses of curcumin (up to 12 g per day) are pharmacologically safe and well tolerated in humans (34). However, unformulated curcumin exhibits very poor bioavailability, with very low concentrations detected in serum and extracellular tissues after oral consumption, likely due to poor absorption, followed by rapid metabolism and elimination (24). Piperine slows the metabolism of curcumin by inhibiting hepatic and intestinal glucuronidation (24). Previous studies have shown that administering curcumin with piperine can increase serum concentrations of curcumin by up to 2000% (35), indicating that glucuronidation inhibition may be the major mechanism of increasing curcumin bioavailability (24). As such, the formulation of the supplement administered in the current study, which contained 35 g of raw turmeric root, (estimated to contain 1,400 mg of curcumin) and 10 mg of piperine, may have helped to increase serum concentrations of curcumin such that it could exert its biological action, whilst the mode of administration may have encouraged greater adherence. These are major strengths of this study, as these factors increase the likelihood of professional athletes utilising this supplement to expedite recovery.

Another strength of this study is the cohort of elite footballers studied, which is rare for this type of research. Whilst research in sub-elite populations can be informative, the contextual factors surrounding elite football are unique, and therefore, findings from sub-elite cannot always be readily extrapolated to elite athletes. Moreover, elite football match-play elicits a high degree of muscle damage and soreness (5), which results in many players using NSAIDs to manage pain and maintain performance amidst an intensive training and match-play schedule (36). However, chronic NSAID use has been associated with various adverse cardiovascular issues (37) and regular use is not recommended (38). As such, findings of the present study in this highly relevant population, provide evidence that a commercially available and easy to administer turmeric shot could reduce muscle soreness and expedite recovery after match-play.

This study is not without limitations. Firstly, despite the supplement being formulated to enhance bioavailability, this has not been empirically tested. Future studies should aim to measure the appearance of curcumin in blood, and the time course, to establish the optimal dosing strategy. Secondly, due to the novelty of the supplement provided and the population, we were unable to blind participants to the treatment they received. Previous research has showed that when participants perceive that they have ingested an active supplement this can distort perceptions of pain (39) and influence other outcomes, such as exercise performance (40). It’s important that future studies incorporate a suitable placebo condition to mitigate this risk. Thirdly, due to the novelty of the population studied, sample size for this study was inherently small, and turmeric and non-turmeric users could not be matched, as we could not control the match-day playing team. This resulted in a larger number of sample points for the supplementation group (60) compared to control (16).

In conclusion, this applied pilot study found that twice-daily consumption of a turmeric supplement attenuated a blood marker of inflammation and subjective muscle soreness, in elite male footballers following match-play. These findings suggest that a commercially available and easy to consume turmeric supplement may accelerate post-match recovery and this warrants further investigation.

## Data availability statement

The raw data supporting the conclusions of this article will be made available by the authors, without undue reservation.

## Ethics statement

The studies involving human participants were reviewed and approved by Nottingham Trent University Human Invasive Ethics Committee (REF: 716). The patients/participants provided their written informed consent to participate in this study.

## Author contributions

DC, RB, and IV: conceptualisation. DC, RB, PH, RJ, and IV: methodology. DC, RB, PH, RJ, CS, SG, RM, and IV: formal analysis. DC, RB, PH, RJ, CS, AC, RS, SG, RM, and IV: data curation, writing—review and editing, and project administration. DC, PH, RJ, CS, and IV: writing—original draft preparation. All authors contributed to the article and approved the submitted version.

## Conflict of interest

The authors declare that the research was conducted in the absence of any commercial or financial relationships that could be construed as a potential conflict of interest.

## Publisher’s note

All claims expressed in this article are solely those of the authors and do not necessarily represent those of their affiliated organizations, or those of the publisher, the editors and the reviewers. Any product that may be evaluated in this article, or claim that may be made by its manufacturer, is not guaranteed or endorsed by the publisher.
