# Supplementary material for: Turmeric supplementation improves markers of recovery in elite male footballers: a pilot study
Source: Front Nutr. 2023 May 24;10:1175622. doi: 10.3389/fnut.2023.1175622 (PMC10244580; doi:10.3389/fnut.2023.1175622)
Supplement: Supplementary file 1 [file Table_1.docx]

**Supplementary materials**

| Group | Playing Time (mins) | Total Distance (m) | High Speed Distance (m) | Acceleration (count) | Deceleration (count) |
| --- | --- | --- | --- | --- | --- |
| Turmeric | 94.2 (13.3) | 9580 (1407) | 642(240) | 29.9 (10.4) | 37.5 (12.9) |
| Non-turmeric | 93 (11.5) | 9825 (1133) | 721 (267) | 30.3 (9) | 40.1 (14) |
| P value | 0.73 | 0.47 | 0.29 | 0.89 | 0.51 |

**Supplementary file 1.** Match-play GPS data for turmeric and non-turmeric groups. Data represents the average (±SD) from eight matches.
